# Oestrogen receptor status and survival in women with BRCA2-associated breast cancer

**DOI:** 10.1038/s41416-019-0376-y

**Published:** 2019-02-06

**Authors:** Kelly Metcalfe, Henry T. Lynch, William D. Foulkes, Nadine Tung, Olufunmilayo I. Olopade, Andrea Eisen, Jordan Lerner-Ellis, Carrie Snyder, Shana J. Kim, Ping Sun, Steven A. Narod

**Affiliations:** 1 Women’s College Research Institute, Toronto, ON Canada; 2 Lawrence S. Bloomberg Faculty of Nursing, University of Toronto, Toronto, ON Canada; 3 Department of Preventive Medicine and Public Health, Creighton University School of Medicine, Omaha, NE Canada; 4 Program in Cancer Genetics, McGill University, Montreal, QC Canada; 5 Beth Israel Deaconess Medical Center, Boston, MA USA; 6 Department of Medicine, University of Chicago, Chicago, IL USA; 7 Toronto Sunnybrook Regional Cancer Center, Toronto, ON Canada; 8 Pathology and Laboratory Medicine, Mount Sinai Hospital, Toronto, ON Canada

**Keywords:** Breast cancer, Mutation

## Abstract

**Background:**

To evaluate the predictors of mortality, including ER status, in women with a BRCA2 mutation and breast cancer.

**Methods:**

Eligible participants were identified from within two longitudinal cohorts. These patients were selected because they were diagnosed with breast cancer between 1975 and 2015 and carried a BRCA2 mutation. Data were abstracted from the medical record and pathology report. We analysed the effects of ER status and other variables on breast cancer specific survival using a Cox proportional hazards model.

**Results:**

Three hundred ninety women with breast cancer and a BRCA2 mutation were included in the analysis. The mean follow-up time was 12.3 years (range 1–39 years) and 89 subjects died (22.8%). In the multivariate analysis, women with ER-positive tumours were more likely to die than women with ER-negative tumours (HR 2.08, 95% CI 0.99–4.36, *p* = 0.05), and this was of borderline significance. For the 233 women with ER-positive tumours the 20-year survival rate was 62.2%, compared to 83.7% for 58 women with ER-negative tumours (*p* = 0.03).

**Conclusions:**

The majority of women with a BRCA2 mutation present with ER-positive breast cancer, and for these women, prognosis may be worse than for BRCA2 carriers with ER-negative breast cancer.

## Introduction

Women with a mutation in BRCA2 have a lifetime risk of developing breast cancer of ~70%.^1^ Approximately two percent of all breast cancers are due to mutations in the BRCA2 gene, and there is emerging evidence that women with a BRCA2 mutation have a worse prognosis compared to women with a BRCA1 mutation or with a sporadic breast cancer.^2,3^ This difference could be attributed to the fact that BRCA2 mutation carriers present with more aggressive tumours compared to women without a mutation,^2^ and BRCA2 oestrogen receptor (ER)-positive breast cancers tend to be luminal B^4^ and have higher oncotype recurrence scores compared to sporadic breast cancers.^5–7^ Alternatively, the BRCA2 mutation could be an independent predictor beyond the pathological characteristics of the cancer.

Women with sporadic breast cancer experience better survival if they present with ER-positive tumours, compared to women with ER-negative tumours.^8,9^ However, it has recently been reported that the opposite relationship is observed in women with BRCA2-associated breast cancers. Jonasson et al. reported that among 285 women with a founder Icelandic BRCA2 mutation, positive ER-status was associated with a greater risk of death, compared to negative ER status.^10^ In that study, after adjustment for other prognostic factors and treatments, women with a BRCA2 mutation had worse long-term survival compared to women without a BRCA2 mutation; this difference was mainly observed in women with ER-positive breast cancer. Their study only included women with a specific BRCA2 mutation (999del5) and it is unclear if the same pattern is observed in women with other BRCA2 mutations. In the current study, we report on the predictors of mortality, including ER status, in women with a BRCA2 mutation and breast cancer.

## Methods

Eligible subjects were identified through merging of two existing cohorts of women with breast cancer and a BRCA2 mutation, including a North American BRCA-associated breast cancer treatment study cohort^11^and an international BRCA risk factor study.^12^ The study sample for the North American cohort study included women with a BRCA2 mutation diagnosed with stage I or stage II breast cancer at age 65 or below, between 1975 and 2008. The international risk factor study includes women with a *BRCA* mutation from 35 centres in nine countries who are followed prospectively from time of genetic testing. These cases included prevalent cases at the time of enrolment and incident cases diagnosed in the follow-up period among women who were cancer free at the time of enrolment.

All study procedures were approved by the institutional review boards at each of the participating centres. For the current study, we included only subjects with BRCA2 mutation who were diagnosed with invasive breast cancer between 1975 and 2015, and for whom a pathology report or treatment record was available. We identified a total of 390 eligible subjects, including 315 from the North American cohort study, and 75 from the international prospective cohort study.

### Study protocol

The treatment records and pathology documents were reviewed. We recorded tumour size (in centimeters), nodal status (positive/negative) and tumour grade (I–III). ER status was recorded as positive, negative, equivocal or unknown. We recorded the use of chemotherapy (yes/no), tamoxifen (yes/no), radiotherapy (yes/no) and bilateral salpingo-oophorectomy (yes/no). Vital status was recorded at time of last follow-up as living or deceased. Vital status was provided by the clinician/investigator affiliated with the centre for the subject by review of hospital medical records and in some cases communication with the next of kin.

### Statistical analysis

A series of survival analyses were performed. The primary endpoint was death. We chose this endpoint because the cause of death was missing in 13 of the 89 women who died. We considered the woman to be at risk for death from the date of the first surgical procedure until the last date of follow-up or until death. Hazard ratios were estimated using the Cox proportional hazards model, implemented in SAS. We evaluated the use of tamoxifen and chemotherapy as dichotomous variables. Oophorectomy was evaluated as a time-dependent variable. We estimated the effect of ER status in the entire patient population and then in subgroups defined by age at diagnosis (<50 or >50). The hazard ratios were adjusted for age of diagnosis, tumour size, lymph node status, chemotherapy, tamoxifen and oophorectomy.

## Results

The characteristics of the 390 subjects with BRCA2-associated breast cancer are presented in Table 1. The women were diagnosed between 1975 and 2015 and were followed for a mean of 12.3 years (range 1–39 years).

**Table 1:** Frequency and mean values for related variables of the 390 subjects with BRCA2 mutations

Variables	Mean or frequency
Date of birth	1950.1 (1899–1978)
Date of diagnoses (range)	1996.1 (1975–2015)
1975–1980	32 (8.2%)
1981–1990	77 (19.7%)
1991–2000	159 (40.8%)
2001–2010	102 (26.2%)
2010–2015	20 (5.1%)
Age at diagnoses (range)	46.0 (24–103)
Years of follow-up (mean) (range)	12.3 (0–39)
Vital status	
Alive	236 (60.5%)
Dead	89 (22.8%)
Lost to follow-up	65 (16.7%)
Cause of death	
Breast cancer	60
Other	16
Missing	13

During the follow-up period, 89 subjects died (22.8%). Of these 60 died from breast cancer and the cause of death was missing for 13 patients. The ten-year breast cancer survival rate for the entire cohort was 83.3%.

**Fig. 1:**
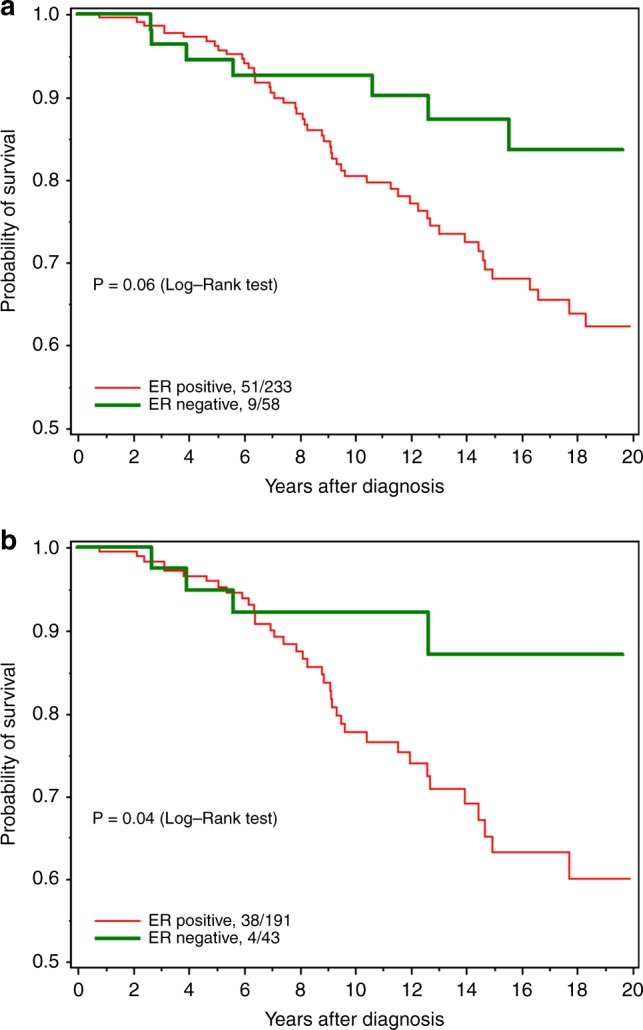
**a** All-cause survival by ER status. **b** All-cause survival for women diagnosed after 1990, by ER status

**Fig. 2:**
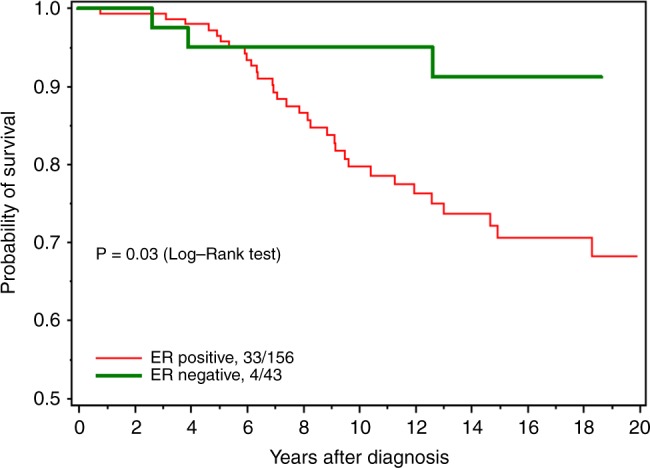
All-cause survival for women under 50, by ER status

Table 2 presents the relative risks of death according to demographic, clinical and treatment variables. The variable that most strongly predicted mortality in women with BRCA2-associated breast cancer was positive ER status; in the multivariate analysis, women with ER-positive tumours were more likely to die than women with ER-negative tumours (HR 2.08, 95% CI 0.99–4.36, *p* = 0.05). Two hundred thirty-three women had ER-positive tumours; for them, the 20-year survival rate was 62.2%, compared to 83.7% in women with ER-negative tumours (*p* = 0.03) (Fig. 1). For women under age 50 at diagnosis (Fig. 2), the survival disadvantage associated with ER-positive tumours as compared to ER-negative tumours was even more profound (20-year survival rate 68.3 vs. 91.3%; *p* = 0.02).

**Table 2:** Relative risk (RR) on death for related variables (all subjects)

Variables	N	N deaths percent read by row	Univariate RR (95% CI), P	Multivariate RR (95% CI)P
Age at diagnosis (years)	390	89 (22.8%)	1.02 (0.99–1.04), 0.15	1.02 (0.99–1.04), 0.23
ER status				
Negative	58 (14.9%)	9 (15.5%)	1	1
Positive	233 (59.7%)	51 (21.9%)	1.94 (0.95–3.95), 0.07	2.08 (0.99–4.36), 0.05
Borderline	9 (2.3%)	0		
Missing	90 (23.1%)	29 (32.6%)		
Mastectomy				
No	169 (43.3%)	33 (19.5%)	1	1
Yes	221 (56.7%)	56 (25.3%)	1.13 (0.73–1.74), 0.59	1.38 (0.68–2.79), 0.37
Grade				
I	23 (5.9%)	3 (13.0%)	1	1
II	96 (24.6%)	20 (20.8%)	1.12 (0.33–3.77), 0.86	1.12 (0.32–3.97), 0.86
III	106 (27.2%)	18 (17.0%)	0.87 (0.26–2.96), 0.82	0.77 (0.21–2.80), 0.69
Missing	165 (42.2%)	48 (29.1%)		
Nodal Status				
Negative	203 (52.1%)	40 (19.7%)	1	1
Positive	145 (37.2%)	39 (26.9%)	1.36 (0.87–2.11), 0.18	0.98 (0.55–1.77), 0.95
Missing	42 (10.8%)	10 (23.8%)		
Size (mm)				
0–10	87 (22.3%)	13 (14.9%)	1	1
11–20	143 (36.7%)	36 (25.2%)	1.37 (0.73–2.59), 0.33	1.65 (0.84–3.23), 0.15
21–30	76 (19.5%)	22 (29.0%)	1.67 (0.84–3.31), 0.14	1.84 (0.85–3.95), 0.12
31+	61 (15.6%)	15 (24.6%)	1.94 (0.92–4.08), 0.08	2.21 (0.95–5.17), 0.07
Missing	23 (5.9%)	3 (13.0%)		
Oophorectomy^a^				
No	123 (31.5%)	46 (37.4%)	1	1
Yes	262 (67.2%)	38 (14.5%)	0.97 (0.78–1.21), 0.80	0.92 (0.72–1.16), 0.45
Missing	5 (1.3%)	5 (100%)		
Tamoxifen				
No	180 (46.2%)	47 (26.1%)	1	1
Yes	148 (38.0%)	32 (21.6%)	0.95 (0.60–1.49), 0.82	0.87 (0.52–1.46), 0.60
Missing	62 (15.9%)	10 (16.1%)		
Radiation therapy				
No	195 (50%)	47 (24.1%)	1	1
Yes	167 (42.8%)	36 (21.6%)	0.94 (0.61–1.45), 0.76	1.38 (0.69–2.73), 0.36
Missing	28 (7.2%)	6 (21.4%)		
Chemotherapy				
No	141 (36.2%)	37 (26.2%)	1	1
Yes	221 (56.7%)	46 (20.8%)	1.05 (0.68–1.63), 0.83	1.00 (0.57–1.74), 1.00
Missing	28 (7.2%)	6 (21.4%)		

^a^Time dependent

Women with ER-negative breast cancers were significantly more likely to have grade III tumours than women with ER-positive breast cancers (*p* < 0.001). There were no significant differences in tumour size (*p* = 0.66) or nodal status (*p* = 0.16) between women with ER-negative breast cancer compared to ER-positive breast cancer.

**Fig. 3:**
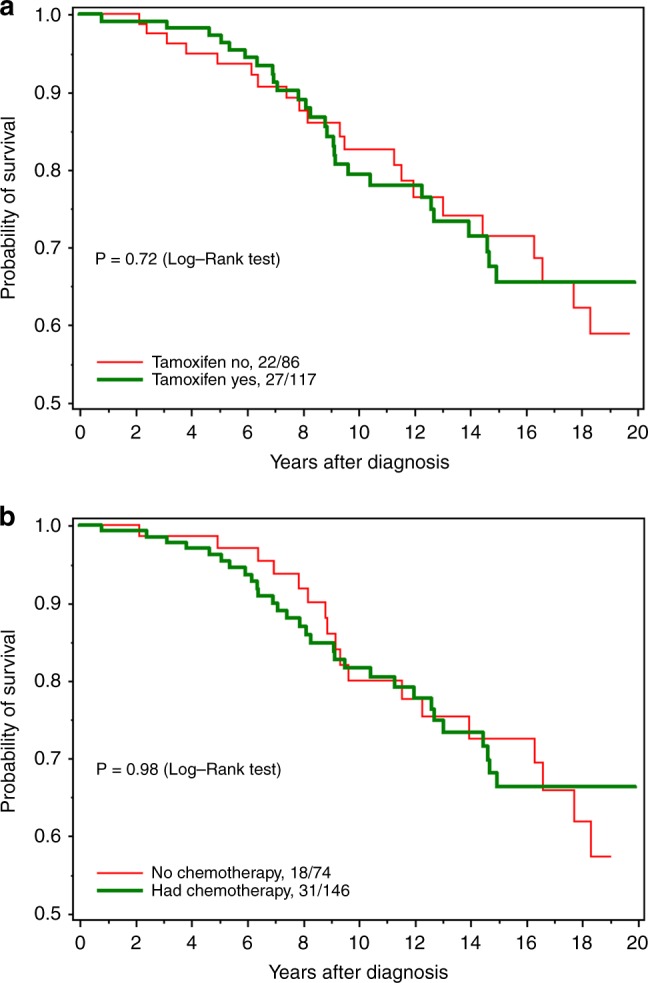
**a** All-cause survival among ER-positive breast cancer, by tamoxifen. **b** All-cause survival among ER-positive breast cancer, by chemotherapy use

Table 3 presents the relative risks of mortality for BRCA2 carriers with ER-positive tumours associated with various treatment variables (including chemotherapy, radiation therapy and tamoxifen). In the multivariate analysis, there was no observed reduction in mortality in BRCA2 mutation carriers with ER-positive breast tumours associated with the use of tamoxifen (HR = 0.91; 95% CI 0.49–1.69, *p* = 0.76) (Fig. 3a) or with chemotherapy (HR = 1.03; 95% CI 0.51–2.06, *p* = 0.94) (Fig. 3b).

**Table 3:** Relative risk (RR) of death for women with ER-positive breast cancer by clinical and treatment variables

Variables	Univariate RR (95% CI), P	Multivariate RR (95% CI), P
Age at diagnosis	1.00 (0.97–1.03), 1.00	1.00 (0.97–1.04), 0.92
Grade		
I or II	1	1
III	0.57 (0.25–1.31), 0.19	0.48 (0.20–1.16), 0.10
Nodal status		
Negative	1	1
Positive	1.24 (0.71–2.18), 0.45	1.02 (0.48–2.14), 0.97
Size (mm)		
0–10	1	1
11–20	1.33 (0.62–2.88), 0.46	1.65 (0.84–3.23), 0.15
21–30	1.41 (0.59–3.37), 0.43	1.84 (0.85–3.95), 0.12
31+	1.04 (0.37–2.93), 0.94	2.21 (0.95–5.17), 0.07
Mastectomy		
No	1	1
Yes	1.05 (0.61–1.83), 0.86	1.27 (0.97–1.04), 0.92
Tamoxifen		
No	1	1
Yes	0.90 (0.51–1.58), 0.72	0.91 (0.49–1.69), 0.76
Radiation therapy		
No	1	1
Yes	1.12 (0.63–1.97), 0.70	1.41 (0.56–3.50), 0.46
Chemotherapy		
No	1	1
Yes	0.99 (0.55–1.77), 0.98	1.03 (0.51–2.06), 0.94
For subjects diagnosed after 1990	1.15 (0.57–2.33), 0.70	1.08 (0.48–2.43), 0.85
Oophorectomy^a^		
No	1	1
Yes	1.10 (0.83–1.45), 0.51	1.18 (0.86–1.62), 0.30

^a^Time dependent

## Discussion

In this international cohort study of 390 breast cancer patients with a BRCA2 mutation, positive ER status was associated with a relatively poor prognosis compared to ER-negative status. Women with ER-positive tumours had a ten-year survival rate of 80.4%, compared to 92.6% in women with ER-negative tumours. After controlling for pathologic features and cancer treatments, positive ER status was the strongest predictor of mortality in this cohort of women with BRCA2-associated breast cancer (HR = 2.08; 95% CI 0.99–4.36, *p* = 0.05). Furthermore, in women with ER-positive tumours, the use of tamoxifen or chemotherapy did not appear to improve survival.

We hereby confirm the findings of Jonasson et al. (2016) who reported that among Icelandic BRCA2 carriers, ER-positive status was an adverse prognostic factor.^10^ Their study included 285 breast cancer patients with a 999del5 BRCA2 mutation, matched with 570 non-carrier patients. After adjustment for various prognostic factors and treatments, a BRCA2 mutation was associated with a significantly worse prognosis than a sporadic breast cancer (HR = 1.61; 95% CI 1.11–2.35, *p* = 0.01). However, this worse prognosis was only observed among women with ER-positive tumours (HR = 1.92, 95% CI 1.20–3.05, *p* = 0.006), and not for women with ER-negative tumours (HR = 1.12, 95% CI 0.54–2.31, *p* = 0.77).

The majority of breast cancers diagnosed in women with a BRCA2 mutation are ER-positive.^2,13^ BRCA2 tumours are significantly more likely to be ER-positive compared to both BRCA1 tumours and sporadic tumours.^13^ We observed this in the current cohort, with 77% of the BRCA2 carriers having ER-positive tumours.

Breast cancer prognostic factors typically include tumour size, nodal status and grade. In the current study of women with BRCA2 mutations, there were no significant differences in mortality associated with these factors. In the adjusted model, mortality was not associated with high grade tumours (HR = 0.77; 95% CI 0.21–2.80, *p* = 0.69), or with positive lymph node status (HR = 0.98; 95% CI 0.55–1.77, *p* = 0.95). The sample size was relatively small and the confidence limits are wide, and it is important that these results be confirmed in other settings.

ER-status has been shown to be an independent favourable predictor of outcome in women with breast cancer.^14^ However, certain subgroups of patients with breast cancer do not experience a favourable prognosis associated with positive ER status, including women diagnosed with breast cancer under the age of 40 years. In a recent analysis of 1910 Canadian women with breast cancer, the prognostic effect of ER-status differed according to age of diagnosis.^15^ Among 213 women diagnosed with breast cancer under the age of 40 years, 15-year survival was significantly worse for women with ER-positive tumours, compared to those with ER-negative tumours (55 vs. 61%). For those diagnosed with breast cancer over the age of 40 years, positive ER status was a favourable prognostic factor.

We have previously reported that oophorectomy reduces the risk of long-term mortality in women with BRCA-associated breast cancer by 54%; however, this benefit was significant for women with a BRCA1 mutation ((HR = 0.38; 95% CI 0.19–0.77, *p* = 0.007), but was not significant for women with a BRCA2 mutation (HR = 0.57, 95% CI 0.23–1.43, *p* = 0.23), although the latter group was small.^11^ Furthermore, the beneficial effect of oophorectomy was only observed in women with ER-negative breast cancers (HR = 0.07; 95% CI 0.01–0.51, *p* = 0.009), and not for women with ER-positive breast cancers (HR = 0.76; 95% CI 0.32–1.78, *p* = 0.53). In the current study, we also did not observe a survival benefit associated with oophorectomy in women with BRCA2-associated breast cancer.

Adjuvant hormone therapy (tamoxifen) is indicated for women with ER-positive tumours, and has been shown to decrease the risk of local recurrence and death.^16–18^ However, there is evidence from several studies that a beneficial effect of adjuvant hormone therapy is not observed in women with BRCA2-associated breast cancer. Goodwin et al. reported that for breast cancer patients that had taken adjuvant hormone therapy, women with a BRCA2 mutation had a higher risk of death compared to sporadic breast cancers (HR = 2.05; 95% CI 1.07–3.91; *p* = 0.03).^2^ In the current study, the use of tamoxifen did not significantly decrease the risk of death in women with ER-positive breast cancer (HR = 0.91; 95% CI 0.49–1.69, *p* = 0.76), in support of the findings of Jonasson et al. (2016) (HR = 1.03; 95% CI 0.41–2.60).

In 285 Icelandic women with BRCA2 mutations, the use of adjuvant chemotherapy decreased the risk of mortality (HR = 0.35; 95% CI 0.16–0.80, *p* = 0.01); this was not observed in (matched) sporadic breast cancers in Iceland (HR = 0.98; 95% CI 0.47–2.04, *p* = 0.96).^10^ In the current study, we observe a significant benefit associated with chemotherapy neither in women with BRCA2-associated breast cancer (HR = 1.00; 95% CI 0.57–1.74, *p* = 1.00), nor in the subgroup of women with ER-positive tumours (HR = 1.03; 95%CI 0.51–2.06; p = 0.94). More research is needed to evaluate the effect of various chemotherapy regimens on survival in women with BRCA2-associated breast cancer, in particular with platinum-based regimens, which have shown preliminary evidence of effectiveness in BRCA1 mutation carriers.^19–22^

The POSH study^23^ followed 137 women with a BRCA2 mutation for death and other clinical outcomes, but there were only 21 ER-negative cases among these. They found that BRCA2-positive status did not negatively impact upon survival, but they were unable to evaluate the effect of ER-status on prognosis on the BRCA2-positive subgroup.

There are several limitations to our study. This is a historical cohort study, so participants were not randomised to various treatments. We did not have the date of genetic testing of these women, and therefore we could not exclude the possibility of survivorship bias (this would occur in study subjects over-represented by long-term survivors); however, there is no reason that this would be different for ER-positive vs. ER-negative cases.

The study was not designed to investigate the effect of various treatments, and it is possible that the women with and without chemotherapy had different risk profiles. Furthermore, women included in this study were diagnosed with breast cancer over a 40-year period (between 1975 and 2015) and treatments have evolved since then, which may have impacted survival estimates. In particular both tamoxifen and chemotherapy were introduced in this timeframe. We do not have the details of the individual chemotherapies but few, if any, patients would have received a platinum-based therapy. ER status was missing in ~20% of the sample, which may reflect the breast cancer diagnoses years, as ER status was not performed as routinely in the 1970s and early 1980s. The cause of death was missing for 13 patients and therefore we chose all-cause mortality as the principal endpoint. However, given the rarity of BRCA2-positive breast cancers (~2% of all breast cancers) it is difficult to construct a large contemporary cohort.

This study adds to the growing evidence of the poor prognosis associated with a BRCA2 mutation and ER-positive breast cancer. The typical breast cancer prognostic factors, including grade, tumour size and nodal status, may not be typical for women with BRCA2 mutations. The observed lack of effective treatments for ER-positive BRCA2-associated breast cancer requires attention, and future research needs to be conducted to evaluate the contribution of newer treatments, including platinum-based chemotherapy, oophorectomy and PARP inhibitors. Moreover, women with a BRCA2 mutation without breast cancer should be counselled on the poor prognosis associated with BRCA2-associated breast cancer when making cancer risk reduction decisions.

## Data Availability

K.M. and S.N. had full access to all of the data in the study and take responsibility for the integrity of the data and the accuracy of the data analysis. Data requests may be submitted to K.M., which will be submitted for ethical approval (kelly.metcalfe@utoronto.ca).
